# Comment on “Is There a Risk of Yellow Fever Virus Transmission in South Asian Countries with Hyperendemic Dengue?”

**DOI:** 10.1155/2015/154146

**Published:** 2015-11-29

**Authors:** John T. Cathey

**Affiliations:** Chom Tian, 414/18 Moo 12, Thappraya Road, P.O. Box 150, Nongprue, Banglamung, Chon Buri 20150, Thailand

I recently came across the interesting article by Agampodi and Wickramage about the possibility of yellow fever transmission into South Asia [1]. The authors provided an excellent review of all the previously postulated hypotheses that have attempted to explain why the disease has never appeared in Asia. However, I think they may have been too quick to dismiss the differences in the volume of the slave trade to the Americas versus that to Asia. I have not undertaken a thorough search on the issue, but from reviewing the Wikipedia entry on “Coolie” in addition to a few freely available sources on the Web, it seems that the “coolie trade,” like the transatlantic slave trade, was mostly one way. In a Ph.D. dissertation, Narvaez writes that the number of the Chinese entering Cuba and Peru was about 125,000 and 92,000, respectively, during the 19th century [2]. He also notes that “Most of the coolies who went from to Cuba and Peru died there. Few ever returned to China or made it to another country because they had no money or did not survive.” According to Wikipedia, the same was true for Indian coolies [3]. The number returning may have been as few as ten percent. Thus, there may not have been “ample opportunities for the introduction of YF to Asia” by means of the coolie trade. The absence of any significant trade in slaves or coolies from Africa or from the Americas to Asia in contrast to that from Africa to the Americas may very well be an important explanation for the mystery of why yellow fever transited the Atlantic, but it has never appeared in Asia [4].
